# The Epidemiology of Arboviral Infections in the Non-monsoon Season: A Clinical and Geospatial Analysis From a Tertiary-Care Setting

**DOI:** 10.7759/cureus.104299

**Published:** 2026-02-26

**Authors:** Dhrubajyoti J Debnath, Vincent Mangayarkarasi, Remya M John, Rakesh U K, Sridhar Amalakanti, Divya P, A Jeganish, Yamini Marimuthu

**Affiliations:** 1 Community and Family Medicine, All India Institute of Medical Sciences, Mangalagiri, Mangalagiri, IND; 2 Clinical Microbiology, All India Institute of Medical Sciences, Madurai, Madurai, IND; 3 General Medicine, All India Institute of Medical Sciences, Mangalagiri, Mangalagiri, IND; 4 Neurology, All India Institute of Medical Sciences, Mangalagiri, Mangalagiri, IND; 5 Community Medicine, Employees State Insurance Corporation (ESIC) Medical College and Post Graduate Institute of Medical Science and Research (PGIMSR), Bangalore, IND; 6 Communicable Diseases and Surveillance, Jawaharlal Institute of Postgraduate Medical Education and Research (JIPMER) International School of Public Health (JISPH) Puducherry, Puducherry, IND

**Keywords:** arboviral infection, chikungunya, dengue, japanese encephalitis, public health surveillance

## Abstract

Introduction: Arboviral infections have a severe impact on human health. The primary objective of our study was to determine the proportion of laboratory-confirmed dengue, Japanese encephalitis, Zika, and chikungunya infections among adults presenting with clinically suspected arboviral illness to a tertiary-care hospital during the non-monsoon season. The secondary objective was to assess the geospatial distribution of confirmed cases. We also aimed to identify factors associated with hospital admission among the confirmed cases.

Methods: A cross-sectional study was done among 507 adult cases with clinically diagnosed arbovirus infection. A pretested questionnaire was used to collect sociodemographic data and clinical features. Blood samples were collected and tested for dengue NS1 antigen, dengue IgM antibody, chikungunya IgM antibody, and Japanese encephalitis IgM antibody by enzyme-linked immunosorbent assay (ELISA). RT-PCR was done to test for Zika virus RNA.

Result: The mean (SD) age of the participants was 38.0 (13.9) years. One hundred fifteen (22.7%) participants had dengue infection, 33 (6.5%) were positive for chikungunya IgM, and six (1.2%) were positive for Japanese encephalitis IgM. However, none tested positive for the Zika virus. Hence, a total of 30.4% of participants had arboviral infection. Gender was found to be statistically associated with hospital admission among the arboviral-positive patients.

Conclusion: A third of clinically suspected adult cases during the non-monsoon period in this hospital-based cross-sectional investigation had laboratory-confirmed arboviral infection, predominantly dengue. The results highlight the continued existence of arboviral infections outside of conventional peak months and support the necessity of continuous monitoring, even though seasonal comparisons and population-level transmission patterns cannot be established.

## Introduction

Arthropod-borne viruses, or arboviruses, are a group of RNA viruses mostly spread to humans and other vertebrates by the bites of infected arthropods, including mosquitoes, sandflies, and ticks. Dengue virus infection (DVI) affects over two-fifths of the global population, with approximately 390 million infections occurring annually. Globally, arboviruses have severe impacts on human health, contributing to as many as 700,000 deaths each year [1-3]. They are endemic to over 100 countries and are mostly seen in tropical and subtropical regions [4]. As per the World Health Organization, the incidence of DVI has increased from 505430 cases in 2000 to 5.2 million in 2019 [5]. Chikungunya, yellow fever, Japanese encephalitis (JE), West Nile, Zika, and tick-borne encephalitis viruses are other arboviruses that cause serious human illnesses. About 70% of the disease burden is in developing countries, particularly in regions like India, Southeast Asia, and South America [6]. Urbanization, climate variability, inadequate waste management, and the expansion of Aedes aegypti and Aedes albopictus habitats are key contributors to this rising burden.

The varied topography and temperature in the Indian subcontinent foster the growth of arthropod vectors, which makes it easier for arboviruses to spread across the nation, resulting in endemicity of DVI, JE, and chikungunya. Factors like urbanization, population growth, and environmental changes further exacerbate the risk of arboviral outbreaks in India. India accounts for a major proportion of global DVI and experiences periodic outbreaks, particularly during the monsoon season when mosquito breeding is more prevalent. According to the National Vector Borne Disease Control Program (NVBDCP) of India, in 2019, there were over 157,000 cases of dengue reported across the country [3,7]. Co-circulation of multiple dengue virus serotypes and increasing serotype shifts have contributed to more severe disease manifestations. Climatic conditions, particularly monsoon and post-monsoon seasons, create favorable breeding habitats for the vector, leading to seasonal surges. The average national seroprevalence is around 49%, while it rises to 77% in the southern part of India [8-10]. About 823,786 people were afflicted with DVI in India between 2018 and 2023, with 1,134 deaths [7]. About 7945 people reported JE during this period, with 786 deaths, and chikungunya affected 51,953 people, with no recorded fatalities [7]. These numbers might be underestimating the true incidence, particularly for dengue, due to underreporting, asymptomatic cases, and limitations in surveillance systems, as highlighted by previous studies [2,11].

Warmer winters can increase the chance of undetected community transmission of arboviral infection during the typically low-incidence season by prolonging vector survival, extending mosquito breeding cycles, and sustaining low-level virus circulation [12-15]. The persistence of breeding habitats in peri-urban regions, overlapping vectors, and temperature fluctuation can lead to a change in transmission patterns. However, fewer studies reported the prevalence of arboviral infections in non-monsoon seasons in India.

Screening for arboviral infection in winter is crucial since reduced estimated prevalence of arboviral illnesses may cause physicians to ignore them, leading to misdiagnosis or delayed treatment. Arboviral testing may not be routinely performed because many winter-time fever episodes are frequently linked to seasonal or respiratory diseases. Due to this, early cases will be overlooked, allowing silent proliferation and abrupt outbreaks when favorable climatic circumstances develop.

Andhra Pradesh had moderate to high endemicity for DVI, no to very low endemicity for JE, and very low to low endemicity for chikungunya in previous years [16]. The primary objective of our study was to determine the proportion of laboratory-confirmed DVI, JE, Zika, and chikungunya infections among adults presenting with clinically suspected arboviral illness to a tertiary-care hospital during the non-monsoon season. The secondary objective was to assess the geospatial distribution of confirmed cases. We also aimed to identify factors associated with hospital admission among the confirmed cases. Laboratory-diagnosed arboviral infection cases can be used to give an estimation of the burden of the disease and provide valuable data for public health planning, resource allocation, early diagnosis, passive surveillance, and reducing mortality [17].

## Materials and methods

Study design and study setting

A cross-sectional study was conducted in a tertiary health-care medical college hospital in Andhra Pradesh from October 2022 to January 2023. The institution caters to a large catchment area covering both rural and urban populations.

Study population and operational clinical case definition of arboviral infection

Adult individuals with fever and/or myalgia, arthralgia, neurological symptoms (e.g., neck stiffness, partial paralysis, or altered mental state), severe headache, maculopapular rash, or hemorrhagic symptoms were considered clinically diagnosed arboviral illness. The study population consisted of clinically diagnosed arboviral illness patients who presented to the tertiary health-care medical college hospital during the study period.

Sample size and sampling technique

The single population proportion formula, n=z²p(1-p)/d², was used for estimating the sample size. In the formula, z is the standard normal deviate at 95% confidence (1.96), p is the anticipated prevalence of arboviral infection among suspected cases, and d is the allowable margin of error. Based on the previous systematic review and meta-analysis, an expected prevalence (p) of 38.3% was assumed [9]. With a precision (d) of 5% and a 95% confidence level, the estimated sample size was calculated to be 363 participants. Considering 10% non-response, the minimum sample size required was 404 participants. A total of 507 cases were clinically diagnosed with arboviral infection during the study period.

Data collection tool

Data were collected using a structured, pretested proforma, which included sociodemographic characteristics, clinical presentation, and laboratory diagnosis. Clinical details such as information on fever, presence of rash, neurological manifestations, symptoms such as sore throat, cough, breathlessness, vomiting, pain in the abdomen, diarrhea, any warning signs, jaundice, and bleeding manifestations were noted. Blood samples were collected at presentation from 507 cases who were clinically diagnosed as having arboviral illness. For diagnosing DVI, participants presenting within five days of symptom onset were tested for NS1 antigen, while those after five days of symptom onset were tested for dengue IgM. Laboratory testing was performed using Indian Council of Medical Research (ICMR)-Central Drugs Standard Control Organization (CDSCO) validated in vitro diagnostic kits. DVI was assessed using NS1 antigen enzyme-linked immunosorbent assay (ELISA) and IgM antibody capture ELISA (MAC-ELISA) kits (ICMR-National Institute of Virology (NIV), Pune, India); chikungunya and JE were detected using ICMR-NIV certified IgM MAC-ELISA kits; and Zika virus was tested by real-time RT-PCR following the ICMR-NIV protocol. All assays met predefined national performance criteria with minimal documented flaviviral cross-reactivity. Relevant investigations, as needed and advised by the treating physician as a part of routine clinical care, were done to clarify the differential diagnosis. All data were recorded prospectively at the time of patient evaluation and verified against laboratory and clinical records to ensure completeness and accuracy.

Statistical analysis

Data were entered into Microsoft Excel (Redmond, WA, USA) and analyzed using Jamovi 2.7.16 [18]. Continuous variables such as age were summarized using mean (SD) or median (IQR), depending on the normality. Categorical variables like gender and clinical features were described using frequencies and percentages. The primary outcome, the proportion of laboratory-confirmed arboviral infections, was calculated with corresponding 95% confidence intervals. Chi-square or Fisher’s exact test was used for checking the level of significance. P < 0.05 was taken as statistically significant.

Geospatial analysis

The Google Geocode function was used to find the coordinates of the residences of the arboviral-positive participants. The shapefile for the Andhra Pradesh district and sub-district, along with coordinates, was imported to QGIS software 3.40 for generating geospatial distribution. The Coordinate Reference System (CRS) was set to Universal Transverse Mercator (UTM) 44N. Nearest neighbor analysis was used to check for clustering of cases. 

Ethical consideration

Ethical approval for this study was obtained from the Institutional Ethics Committee of All India Institute of Medical Sciences, Mangalagiri (approval AIIMS/MG//IEC/2022-23/196). The study adhered to the ethical principles of the Declaration of Helsinki and the ICMR national guidelines for biomedical research [19]. Informed consent was obtained from the study participants. Confidentiality of participant information was maintained.

## Results

Among the 507 participants with clinical presentation of arboviral infection, the mean (SD) age was 38.0 (13.9) years. The majority belonged to the 18-30 years age group (38.1%), followed by those aged 31-45 years (33.9%), 46-60 years (19.3%), and 61-75 years (8.1%). Participants aged older than 75 years constituted 0.6% of the study population. About 267 (52.7%) were female and 240 (47.3%) were male. Most participants were residents of Guntur district (66.7%), followed by Krishna district (24.3%). Details regarding the sociodemographic characteristics are given in Table 1.

**Table 1:** Distribution of participants according to sociodemographic characteristics (n=507)

Sl no	Characteristic	frequency	(%)
1	Age category		
	18-30 years	193	(38.1)
	31-45 years	172	(33.9)
	46-60 years	98	(19.3)
	61-75 years	41	(8.1)
	More than 75 Years	3	(0.6)
2	Gender		
	Male	240	(47.3)
	Female	267	(52.7)
3	District		
	Guntur	338	(66.7)
	Krishna	123	(24.3)
	Prakasam	13	(2.6)
	West Godavari	6	(1.2)
	Khammam	5	(1.0)
	Srikakulam	5	(1.0)
	East Godavari	4	(0.8)
	Nellore	4	(0.8)
4	Hospital Admission		
	Yes	122	(24.1)
	No	385	(75.9)

**Figure 1:**
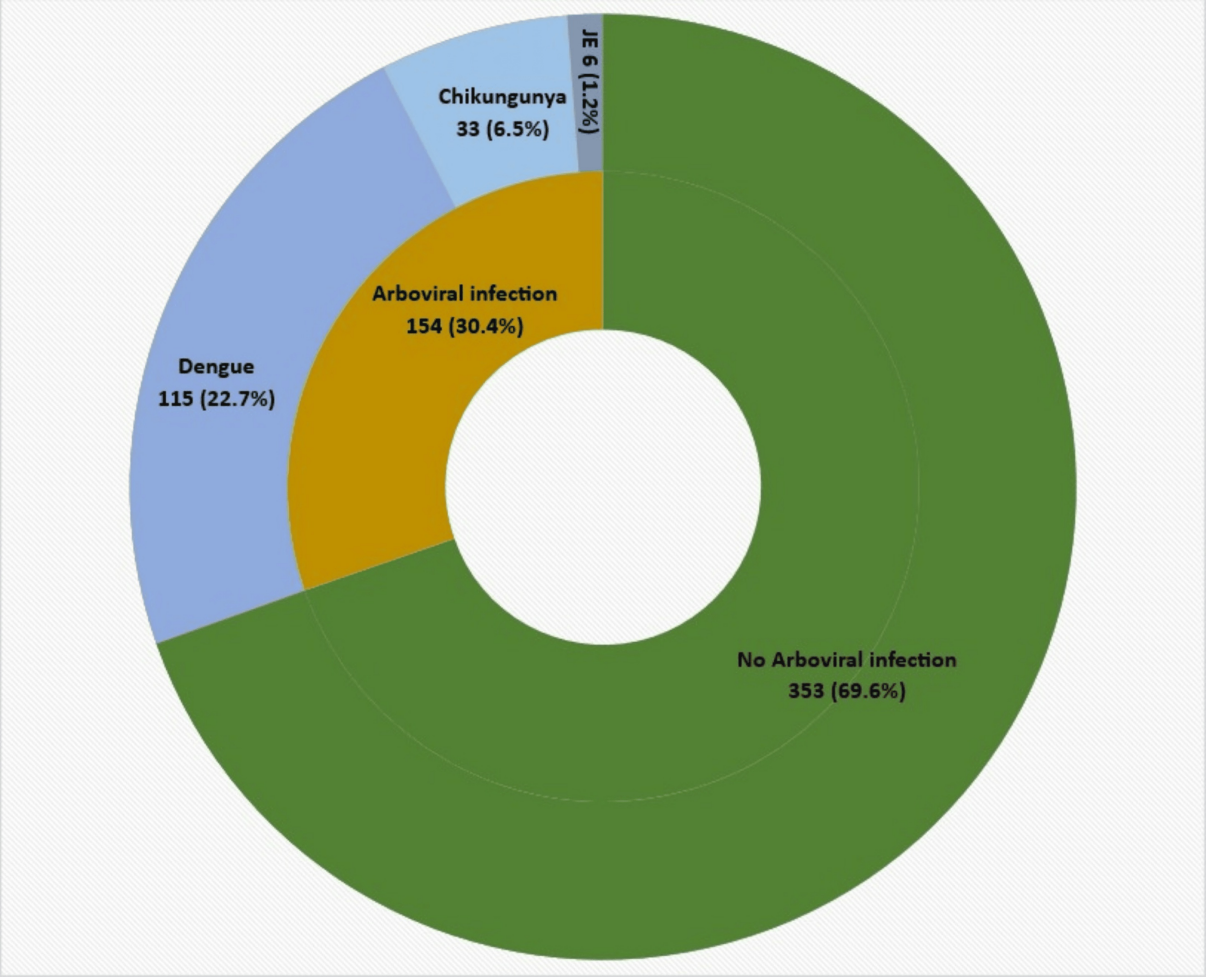
Proportion of dengue, chikungunya and Japanese encephalitis (JE) among the participants (n=507)

Twenty-one (4.1%) participants tested positive for dengue NS1 antigen, while 94 individuals (18.6%) were positive for dengue IgM ELISA. Hence, about 22.7% of the participants had DVI. Thirty-three (6.5%) were positive for chikungunya IgM, while six (1.2%) individuals were positive for JE IgM. However, none tested positive for Zika virus. One hundred fifty-four individuals (30.4%) tested positive for one arboviral infection. Out of 154 laboratory-confirmed arboviral infection cases, 38 (24.7%) had severe manifestations, necessitating hospitalization. The distribution of arboviral infection among participants is depicted in Figure 1.

**Figure 2:**
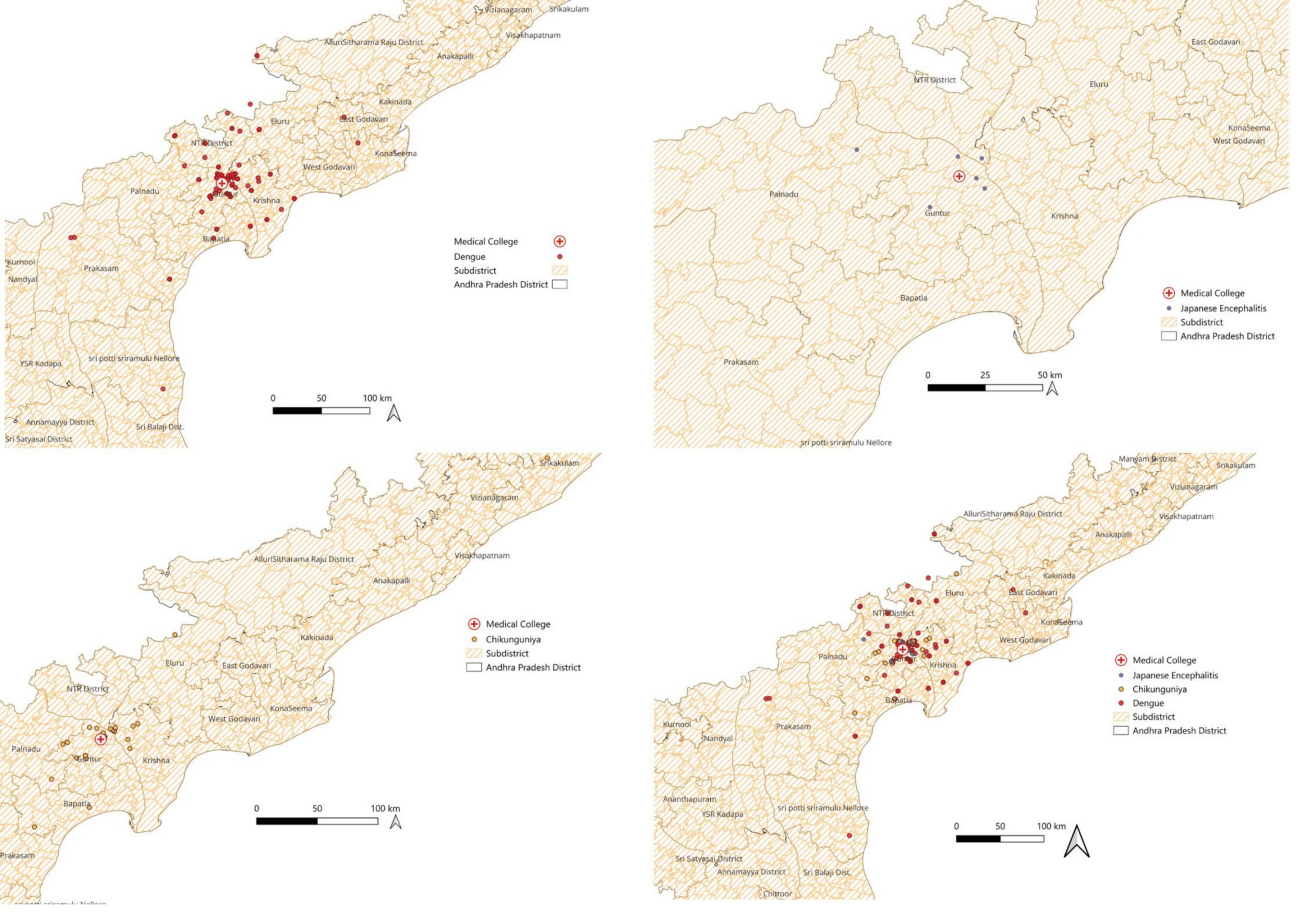
Distribution of A) only dengue infection B) only Japanese encephalitis C) only chikungunya and D) combined dengue, chikungunya, and Japanese encephalitis cases across the districts of Andhra Pradesh

The dengue IgM positivity increased as the age advanced, and it was highest among patients aged 45-60 years (74.2%) and ≥60 years (75.0%), as described in Table 2. It was found that the proportion of dengue IgM positivity was significantly higher in the age group ≥45 years as compared to 18-45 years (p=0.02). Dengue NS1 positivity was higher in the 18-to-30-year-old group (21.8%). NS1 positivity became lower as age advanced (45-60 years (3.2%), ≥60 years (0%), p=0.03). JE IgM was occasionally found between 18 and 45 years, with no cases reported in the 45-to-60-year age range. Chikungunya IgM seropositivity ranged from 19% to 25% across age groups. The distribution of dengue, chikungunya, and JE across the districts of Andhra Pradesh is depicted in Figure 2. The nearest neighbor index (NNI) for DVI was 0.41 (z = −12.17, p < 0.001), and for chikungunya it was 0.57 (z = −4.69, p < 0.001). The NNI for JE was greater than one. 

**Table 2:** Age-wise distribution of arboviral infections among adults (N = 154)

Age group (in years)	Dengue NS1 positive n (%)	Dengue IgM positive n (%)	Chikungunya IgM positive n (%)	Japanese Encephalitis IgM positive n (%)
18–30	12 (21.8)	29 (52.7)	12 (21.8)	2 (3.6)
30–45	8 (15.4)	30 (57.7)	10 (19.2)	4 (7.7)
45–60	1 (3.2)	23 (74.2)	7 (22.6)	0 (0.0)
≥60	0 (0.0)	12 (75.0)	4 (25.0)	0 (0.0)
p-valueᵃ	0.034	0.144	0.94	0.36

**Figure 3:**
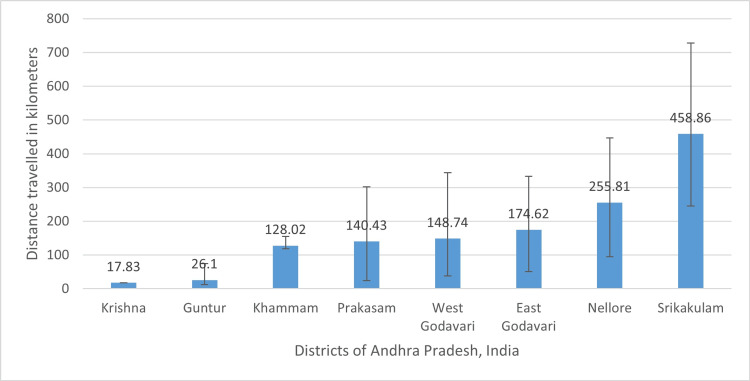
Median (IQR) distance travelled by participants grouped by their districts

The participants travelled a median (IQR) distance of 26.6 (35.5) km. The median (IQR) distance for the various geographic coordinates for the participants within the district in which the hospital is located and outside this district is depicted in Figure 3.

Of the 154 arbovirus-positive individuals, 80 (51.9%) were female and 74 (48.1%) were male. Compared to male patients, the odds of female patients being treated as outpatients (OPD) were significantly higher (OR=2.26; 95% CI, 1.06-4.81; P=0.032). However, there was no statistically significant association found between gender and arboviral seropositivity, such as dengue IgM (P=0.699), dengue NS1 (P=0.608), chikungunya IgM (P=0.653), or JE IgM (P=0.352). Similarly, clinical manifestations such as encephalitis/meningitis, hemorrhagic characteristics, diarrhea, respiratory symptoms, or jaundice did not significantly differ between genders (all P>0.05) (Table 3).

**Table 3:** Association of gender with severity, arboviral positivity, and clinical presentation (N = 154)

Variable/Outcome	Male n (%)	Female n (%)	Odds ratio (95% CI)	p-value
Patient type				
In-patient	24 (32.4)	14 (17.5)	Reference	
Out-patient	50 (67.6)	66 (82.5)	2.26 (1.06–4.81)	0.032
Arbovirus infection positivity				
Dengue IgM positive	44 (59.5)	50 (62.5)	0.88 (0.46–1.68)	0.699
Dengue NS1 positive	9 (12.2)	12 (15.0)	0.79 (0.31–1.99)	0.608
Chikungunya IgM positive	17 (23.0)	16 (20.0)	1.19 (0.55–2.58)	0.653
Japanese Encephalitis IgM positive	4 (5.4)	2 (2.5)	2.23 (0.40–12.5)	0.352
Clinical presentation				
Encephalitis/Meningitis	1 (1.4)	1 (1.3)	1.08 (0.07–17.6)	0.956
Hemorrhagic fever	14 (18.9)	16 (20.0)	0.93 (0.42–2.08)	0.866
Diarrhoea	6 (8.1)	10 (12.5)	0.62 (0.21–1.79)	0.372
Respiratory symptoms	15 (20.3)	15 (18.8)	1.10 (0.50–2.45)	0.812
Jaundice	1 (1.4)	3 (3.8)	0.35 (0.04–3.46)	0.35

Of the 154, 116 (75.3%) received treatment as OPD and 38 (24.7%) were admitted as inpatients (IPD); the difference was statistically significant (p<0.001 (χ² Goodness of Fit)), and dengue IgM positive was higher among OPD (64.7%) than IPD (50%) (Table 4).

**Table 4:** Association of hospital admission with arboviral positivity and clinical presentation (N = 154) ᵃ Odds ratios derived from binomial logistic regression comparing inpatient (IPD) versus outpatient (OPD) status. ^b^ Haldane–Anscombe correction applied due to zero cell count.

Variable/Outcome	Hospital Admission		Odds ratio (95% CI)ᵃ	p-value
	Yes n (%) (n = 38)	No n (%) (n = 116)		
Arbovirus infection positivity				
Dengue IgM positive	19 (50)	75 (64.7)	0.55 (0.26–1.15)	0.11
Dengue NS1 positive	6 (15.8)	15 (12.9)	1.26 (0.45–3.53)	0.656
Chikungunya IgM positive	11 (28.9)	22 (19)	1.74 (0.75–4.04)	0.193
Japanese Encephalitis IgM positive	2 (5.3)	4 (3.4)	1.56 (0.27–8.85)	0.616
Clinical presentation				
Encephalitis/Meningitis	2 (5.3)	0 (0.0)	16.0 (0.75–340)^b^	0.013
Hemorrhagic fever	7 (18.4)	23 (19.8)	0.91 (0.36–2.33)	0.849
Diarrhoea	3 (7.9)	13 (11.2)	0.68 (0.18–2.52)	0.762
Respiratory symptoms	5 (13.2)	25 (21.6)	0.55 (0.20–1.56)	0.347
Jaundice	1 (2.6)	3 (2.6)	1.02 (0.10–10.09)	1.00

## Discussion

This study was done among 507 clinically diagnosed arboviral infection visiting a tertiary care medical college hospital. Among the 507 participants in this study, women were slightly more prevalent (52.7%). This is consistent with studies conducted in the northern region of India but in contrast to a number of regional studies conducted in coastal Karnataka, Mysuru, and Central India, which reported a male majority [20-22]. These variances might be related to geographical variations in healthcare-seeking behaviours, occupational exposure to mosquito bites, and clothing patterns. The age group 18-30 years was the highest age group (38.1%) involved. This is similar to other studies from Mangalore, coastal Karnataka, and Maharashtra, reflecting an increased likelihood of arbovirus infection involving the productive working-age group [20-22].

In our study, 22.7% (115/507) were dengue positive, which was the most common arboviral infection. Dengue IgM was detected in 18.5% and chikungunya IgM in 6.5% of the arboviral-positive cases. This is in accordance with the national and regional data that dengue serotypes are in wide circulation across countries in comparison to other arboviruses [23,24]. The seroprevalence of dengue varies from 35-38% across northern and eastern parts of India to 11-14% in southern and western parts of India [25].

In this study, the NNI for DVI and chikungunya was observed to be less than one, suggesting that the cases were spatially clustered in a statistically significant manner (p < 0.001). The clustering of positive cases around the Guntur and Krishna districts might be because of the proximity of this health care facility in the region, leading to available and affordable health care. The observed geospatial clustering may not accurately reflect the intensity of transmission at the community level, but rather healthcare accessibility, referral pathways, and population density. Since this is a facility-based study, spatial patterns should be interpreted cautiously and not assumed to represent community-level incidence. Only six (1.2%) cases of JE were reported in this study and had an NNI of more than two. This coincides with findings from other studies, which suggest that JE cases were mostly sporadic in adults more than 18 years [26].

In this study, there were no Zika-positive cases detected. However, the absence of detected cases does not exclude low-level circulation. This is in line with studies from Kashmir that reported zero cases of Zika but a high prevalence of dengue (32.7%) and chikungunya (9.6%) [27]. This substantiates that Zika virus transmission is still restricted to particular areas only, although dengue and chikungunya are more prevalent.

This cross-sectional study conducted in a tertiary care hospital shows the percentage of laboratory-confirmed infections among participants who were clinically diagnosed. However, they do not represent prevalence or incidence at the community level. But laboratory-confirmed cases in the non-monsoon period suggest that surveillance systems would benefit from continuing to remain vigilant outside of the conventionally acknowledged peak seasons. Also, direct seasonal inference is limited by the lack of a concomitant monsoon-season comparison. Consequently, it is important to use caution when drawing inferences about shifting transmission patterns, and population-based and longitudinal data are needed for future policy implications.

The positive arboviral cases identified from faraway districts might reflect the healthcare-seeking behavior and willingness to travel long distances to a tertiary care hospital, even though other healthcare facilities may be available nearby. It was found in the present study that the hospital, being a tertiary care treating center, nearly one-third (30.5%) of people were traveling approximately more than 25 kilometers to seek healthcare, even though there exist primary and secondary levels of healthcare at a much lesser distance. The importance of this distance traveled by the patient lies in the fact that while analyzing the data for disease surveillance purposes, the data of the entire state needs to be analyzed to know the exact number of cases district-wise. This holds special significance in those diseases when a few cases can give rise to a potential epidemic. The purpose of geospatial mapping of cases at the state level is thus a very important step towards disease surveillance.

This study found that as age increased, dengue NS1 seropositivity decreased, whereas it was just the opposite for dengue IgM antibody detection, i.e., as age increased, the prevalence of dengue IgM antibody also increased. The NS1 antigen is usually evident early in the disease, and IgM seroconversion occurs later. This is in concurrence with similar studies done in the western part of India [28]. Younger patients typically present early in the illness with highly viremic infections, while the older patients tend to present slightly later in the disease. In this study, chikungunya IgM positivity was distributed across adult age groups without significant variation, while JE IgM was infrequent and observed only in a limited age range between 18 and 45 years, consistent with its lower endemicity and focal transmission in many regions.

There was no seropositivity correlation among the nearly equal gender distribution of 154 arbovirus positives (51.9% female). Studies that reported a significant association between male gender and arboviral infection positivity might be because of occupational exposure or dressing patterns [29]. In this study, females showed increased outpatient management (OR=2.26; 95% CI, 1.06-4.81; P=0.032), which may reflect care-seeking behavior or milder symptoms.

Strength of the study

The study provides recent epidemiological data on the prevalence of arboviral diseases in the study area, which provides essential information for regional public health planning. This study was done during the non-monsoon timeline, drawing attention to the effects of changing climatic patterns in the relatively cooler months. Geographic Information System (GIS)-based spatial mapping of the case distribution made it possible to see hotspots and clusters, which can direct resource allocation and focused vector management. Instead of concentrating on a single pathogen, the inclusion of common arboviral infections (such as dengue, chikungunya, JE, and Zika virus) provided a holistic outlook. The prevalence of JE infection in certain areas provides information regarding the endemicity of this disease, and vaccination programs for this infection can be designed accordingly.

Limitations of the study

Since this study was conducted in a tertiary care hospital, the mild cases might not have come seeking healthcare and, therefore, may have been missed from this study. The study findings are from a single center and involve an adult population, which might affect the generalizability of the findings. Because the study was conducted at a tertiary care facility, its conclusions might not accurately represent population-level transmission dynamics, but rather referral trends, accessibility, and healthcare-seeking behavior will have an impact on hospital-based data, which could lead to selection bias. Lastly, generalizability to pediatric groups is limited since the study findings were in adults.

## Conclusions

A third of clinically suspected adult cases during the non-monsoon period in this hospital-based cross-sectional investigation had laboratory-confirmed arboviral infection, predominantly dengue. The results highlight the continued existence of arboviral infections outside of conventional peak months and support the necessity of continuous monitoring, even though seasonal comparisons and population-level transmission patterns cannot be established.
